# A Phantom-Based Curriculum for Teaching and Assessing Ultrasound Neuraxial Skills in Pediatric Anesthesia Trainees: Development and Implementation

**DOI:** 10.7759/cureus.98807

**Published:** 2025-12-09

**Authors:** Leah Webb, Melissa M Masaracchia, Kim M Strupp

**Affiliations:** 1 Pediatric Anesthesiology, Children's Hospital Colorado, University of Colorado Anschutz Medical Campus, Aurora, USA; 2 Pediatric Anesthesiology, Cohen Children's Medical Center, Zucker School of Medicine at Hofstra/Northwell, Queens, USA

**Keywords:** epidural training, pediatric anesthesiology, simulation in medical education, skills and simulation training, spinal analgesia

## Abstract

Introduction

Opportunities to practice ultrasound-guided/assisted (USGA) neuraxial techniques in pediatric patients are limited due to the high-risk nature and anatomical variability of this population. Simulation-based education offers a safe and effective method for trainees to develop competence in these challenging procedures.

Methods

We developed a curriculum using a previously validated, shelf-stable pediatric spine phantom designed to replicate sonographic anatomy. Pediatric anesthesiology trainees participated in a structured workshop that included pre-workshop online content, a hands-on scanning session, and a three-minute skills assessment. Participants completed identical pre-, post-, and six-month or more follow-up quizzes consisting of 14 knowledge-based questions and items assessing comfort and practice behavior. Paired-samples t-tests were used to compare mean percent correct scores between pre- and post-intervention and between pre- and follow-up assessments. To control for multiple comparisons, a Bonferroni correction was applied (adjusted α=0.025). Analyses were verified using R software (version 4.4.0). Descriptive statistics evaluated shifts in comfort and behavior.

Results

Twenty-six trainees (13 fellows, 13 residents) enrolled; 20 completed the full curriculum, and 18 completed follow-up assessment. Participants demonstrated significant improvement from pre- to post-workshop (*t*(27)=−5.72, *p*<.001, *d*=1.15, large), with sustained knowledge retention at follow-up (*t*(27)=−4.15, *p*<.001, *d*=0.63, medium); similar trends were observed among fellows and residents, indicating consistent learning gains across training levels. All workshop attendees demonstrated proficiency in obtaining six critical neuraxial ultrasound views. Self-reported comfort and frequency of ultrasound use increased across all groups, with positive trends sustained at follow-up. Participant feedback was uniformly favorable.

Conclusions

A phantom-based, simulation-enhanced curriculum significantly improved and sustained pediatric regional anesthesia knowledge, technical skills (based on end-of-workshop assessment), comfort, and reported use of ultrasound for neuraxial procedures in pediatric anesthesia trainees, demonstrating durable learning gains and strong educational impact. This low-risk, reproducible educational model may help institutions implement structured training in USGA neuraxial techniques and promote broader adoption of ultrasound in pediatric regional anesthesia. Future multicenter validation should evaluate its generalizability across diverse training environments.

## Introduction

Despite the increasing use of ultrasound for regional and neuraxial anesthesia, significant training gaps persist in pediatric anesthesia education worldwide. A recent international survey identified that fewer than half of anesthesia trainees perform pediatric regional techniques independently, with most reporting limited exposure to infants and high reliance on direct supervision throughout training [1]. Similar studies have noted that formal, structured curricula for pediatric regional anesthesia remain uncommon, and opportunities for supervised, hands-on ultrasound experience are inconsistent across programs [1,2]. Simulation-based education using validated phantoms therefore offers an important strategy to standardize instruction and ensure competency in these high-risk, low-frequency procedures [2-8].

Pediatric spinal anatomy differs substantially from adults, with features such as less ossification, a lower conus medullaris, higher cerebrospinal fluid turnover, and less dense ligaments [9]. These differences make neuraxial procedures more technically challenging, and the utility of ultrasound for block placement in children has been well established, with evidence of improved safety and success [10-16]. The benefits of ultrasound are even more pronounced with atypical spinal anatomy in syndromic children [17], who comprise a large subset of pediatric surgical patients. Honing pediatric patient-related ultrasound skills for high-risk procedures in a simulation setting is an ideal scenario for learning without exposing patients to unnecessary risk.

We previously demonstrated how to create a cost-effective, reproducible, shelf-stable phantom spine model that generated ultrasound imaging reflective of that seen in actual pediatric patients and that can be used to practice procedures involving the pediatric neuraxis without risk [18]. To evaluate the effectiveness of using our phantom model for education, we designed an ultrasound-guided/-assisted (USGA) neuraxial workshop curriculum for pediatric anesthesiology trainees and assessed their resultant knowledge and skill acquisition.

## Materials and methods

This study was deemed exempt by the Colorado Multiple Institutional Review Board.

As previously described, we developed a spine phantom capable of producing the key ultrasound views relevant for the performance of neuraxial USGA procedures [18]. To evaluate knowledge, skill acquisition, comfort, and practice behavior, using this phantom we developed a three-phase educational workshop: pre-workshop preparation, in-person instruction and practice, and post-workshop evaluation. Participation in this workshop was completely voluntary with no funding or incentive for participation.

Quiz development and use

**Figure 1:**
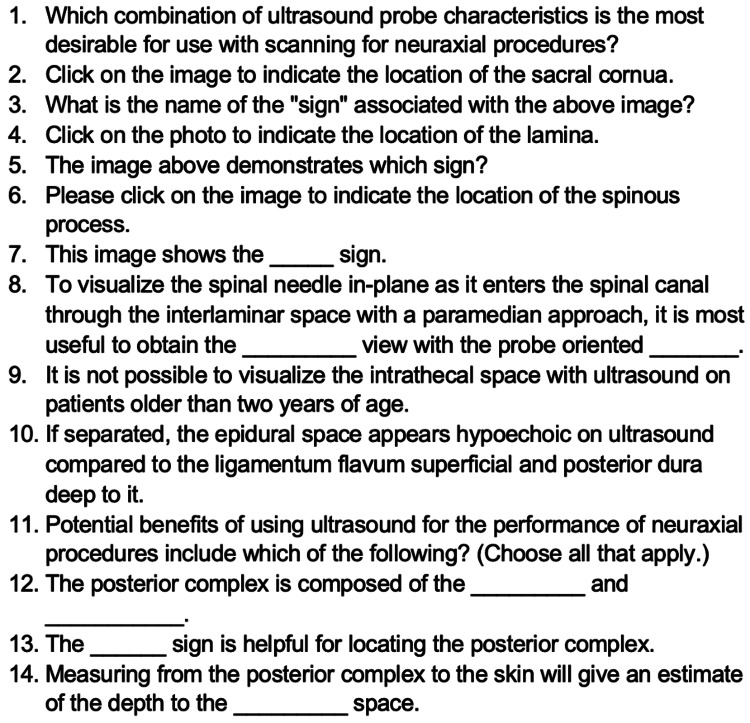
Quiz questions, 14 total, that comprised the knowledge assessment portion in each of the three quizzes (pre-, post-, and follow-up).

A knowledge-based quiz was created to assess understanding of neuraxial ultrasound concepts, including probe selection, image interpretation, and identification of key spinal landmarks. Quiz items were developed in alignment with the educational objectives of the workshop. To establish content validity, two pediatric anesthesiologists with expertise in ultrasound-guided neuraxial anesthesia reviewed the quiz for clarity, accuracy, and alignment with objectives. Revisions were made based on their feedback. The quiz was administered immediately before and after the workshop, and at six, or more, months after to measure change in knowledge. Each assessment consisted of the same 14 multiple-choice and fill-in-the-blank knowledge-based questions (see Figure 1). “Pre”, “Post”, and “Follow Up” quizzes also included Likert-scale items evaluating the current behavior (use of ultrasound for neuraxial procedures), comfort with ultrasound, and/or general feedback on workshop expectations.

Pre-workshop phase

Prior to the workshop, participants completed an anonymous baseline assessment (“Pre-Quiz”). To facilitate preparation, participants were provided with pre-workshop resources, including two instructional spine scan videos and a reference article [19].

In-person workshop phase

**Figure 2:**
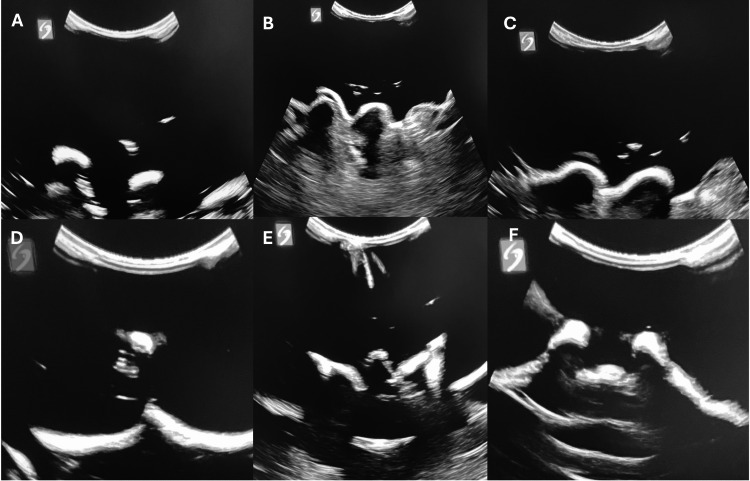
Six critical ultrasound views for neuraxial procedures. Parasagittal (A, B, C) and transverse (D, E, F) images from previously described phantom. A: Parasagittal transverse process view (“trident” sign). B: Parasagittal articular process view (“camel hump” sign). C: Parasagittal oblique interlaminar view (“horse head” or “sawtooth” sign). D: Transverse midline spinous process view. E: Transverse interspinous (interlaminar) view (“bat” or “bat wing” sign). F: Transverse sacral cornua view (“frog” or “frog eye” sign).

**Figure 3:**
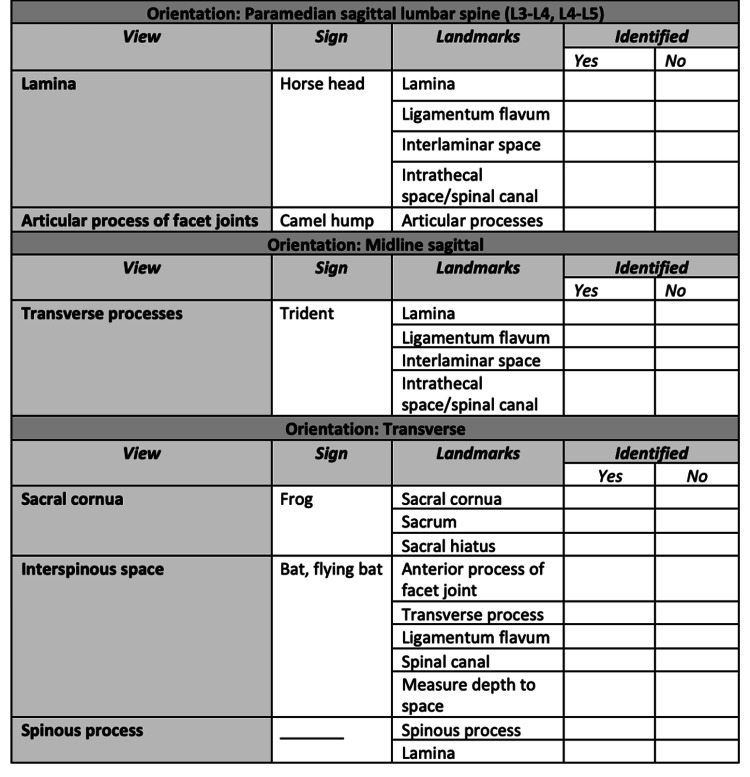
Qualitative assessment tool used by evaluators to track participants’ ability to identify all specified landmarks associated with each neuraxial ultrasound view.

The workshop began with a 15-minute didactic session. This included a slide presentation detailing acquisition of six critical ultrasound views (Figure 2), comprising the five views described in the scanning protocol of Ghosh et al. [19] and one additional transverse sacral cornua view. Frequently missed questions from the pre-quiz were reviewed, followed by a brief (three-minute) live demonstration on a phantom to illustrate acquisition of the six views. Participants then engaged in 30-60 minutes of hands-on practice at multiple stations, with an instructor-to-learner ratio of 1:2-4. Practice was unstructured, allowing learners to independently acquire the six views. Each participant subsequently underwent a three-minute scanning assessment, during which they demonstrated image acquisition of the six critical views and relevant landmarks. Instructors evaluated the performance using a qualitative assessment tool (Figure 3) that measured the ability to identify specific landmarks associated with each view. To ensure consistency among raters, all instructors participated in a brief calibration session prior to the assessments. During this session, they jointly reviewed representative phantom images and agreed upon the criteria defining successful acquisition of each of the six critical neuraxial ultrasound views. The qualitative assessment tool provided explicit guidance for identifying relevant landmarks, thereby standardizing performance evaluation across raters.

Post-workshop phase

Following the intervention, participants completed an anonymous “Post-Quiz”. At six or more months after the workshop, participants completed a similar “Follow-up Quiz”. While the knowledge-based questions were consistent across quizzes, the comfort and behavior, as well as feedback, questions were somewhat varied.

Assessment outcomes

To assess the effectiveness of the neuraxial workshop using custom phantom spine models, pediatric anesthesiology trainees were recruited from April 2022 through December 2024. A priori power analysis was conducted for a two-tailed, paired-samples t-test with an alpha level of 0.05, a desired power of 0.80, and an anticipated large effect size (Cohen’s d=0.8). Based on these parameters, a minimum of 15 participants was required to detect a statistically significant pre- to post-intervention difference. Paired-samples t-tests were used to compare knowledge questions’ mean percent correct scores between pre- and post-intervention and between pre- and follow-up assessments. To control for multiple comparisons, a Bonferroni correction was applied (adjusted α=0.025). Statistical analyses were verified using R software (version 4.4.0; R Foundation for Statistical Computing, Vienna, Austria). Behavior and comfort-related responses were evaluated for directional shifts from less favorable (e.g., “strongly disagree,” “disagree,” “never,” “rarely”) to more favorable categories (e.g., “agree,” “strongly agree,” “frequently,” “always”). Feedback items were assessed for the proportion of participants reporting a positive perception (e.g., “agree,” “strongly agree,” “adequate,” “somewhat adequate”).

## Results

**Figure 4:**
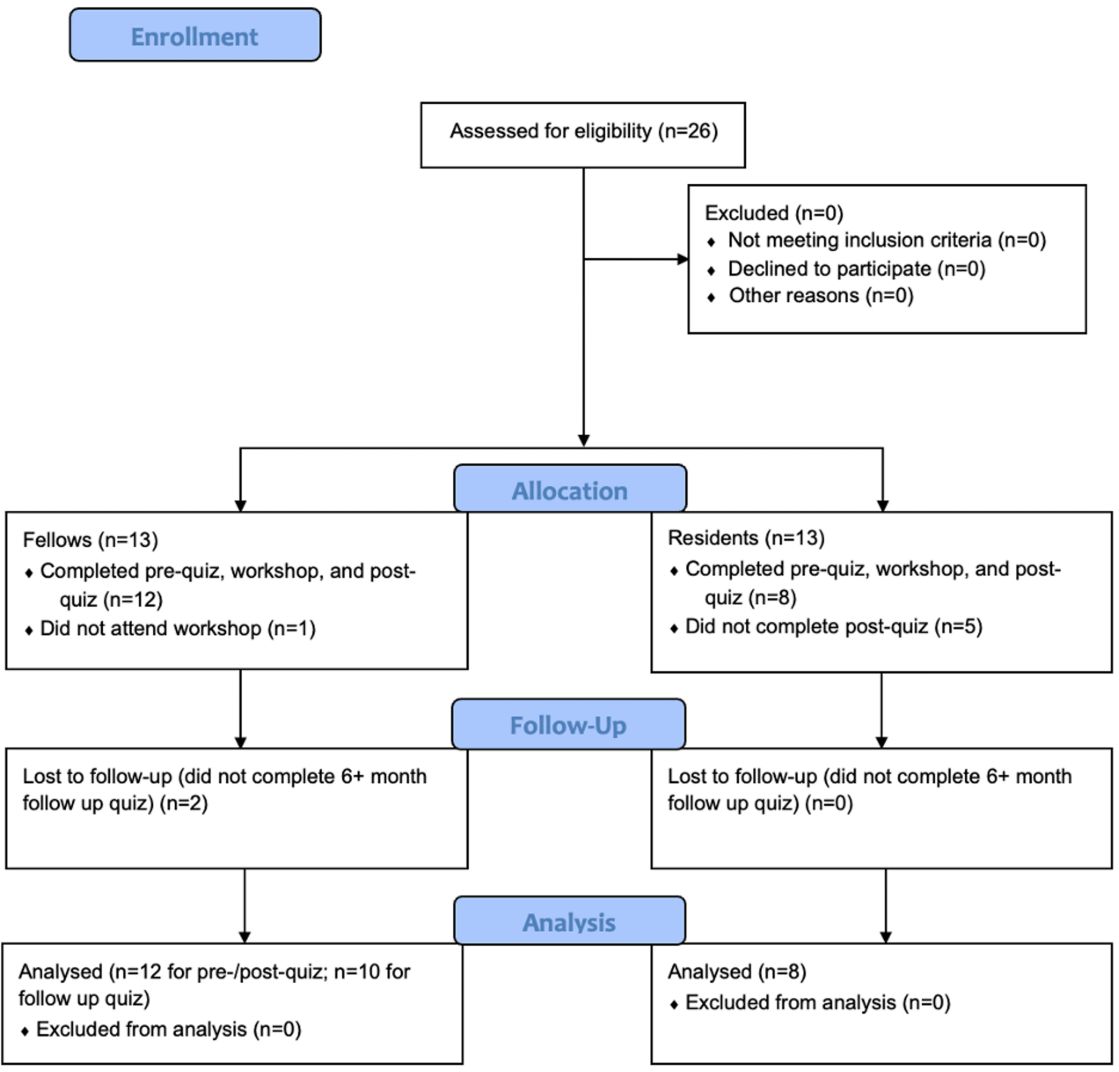
Consolidated Standards of Reporting Trials (CONSORT) flow diagram showing movement of trainees through each step of the study.

**Figure 5:**
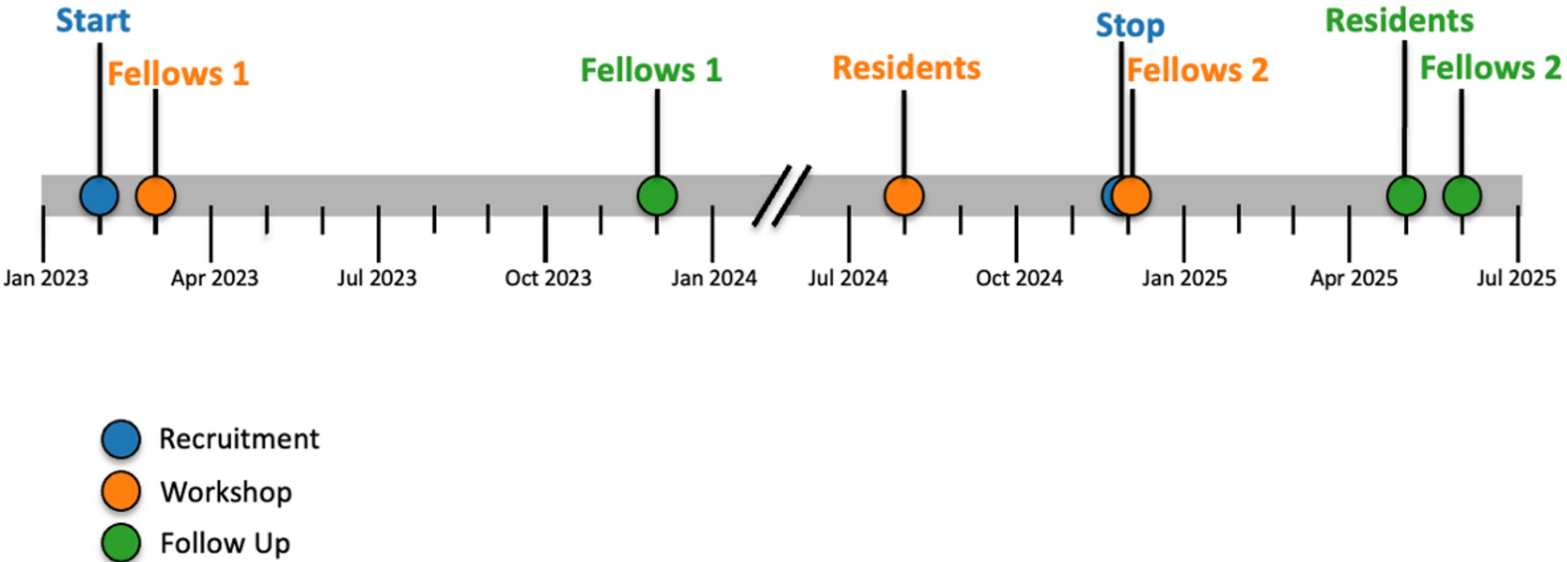
Timeline demonstrating dates of recruitment, workshops, and follow-up period for each cohort. Created with the help of ChatGPT by Dr. Leah Webb.

A total of 26 learners (13 fellows and 13 residents) voluntarily enrolled in the study and completed the pre-intervention quiz. Because this was a voluntary educational study with no funding or incentives for participation, not all trainees completed every phase of the study. Of the 26 who enrolled, 20 (12 fellows and eight residents) completed the full workshop curriculum, including submission of the post-intervention quiz, and were included in the primary analysis. One fellow was unable to attend the live workshop and therefore did not complete the curriculum; their pre-quiz data were excluded from the analysis. Additionally, five residents attended the workshop but did not complete the post-quiz; thus, their pre-quiz data were also excluded. Of the 12 fellows who completed the workshop, two did not submit the follow-up quiz; however, because they had completed both pre- and post-quizzes, their data results were retained in the analysis. Thus, the analysis of the follow-up quiz included the remaining 18 learners (10 fellows and eight residents) who completed the follow-up quiz. These results are summarized in Figure 4 (Consolidated Standards of Reporting Trials (CONSORT) diagram). Figure 5 shows the dates of recruitment, workshop rounds, and follow-up.

**Figure 6:**
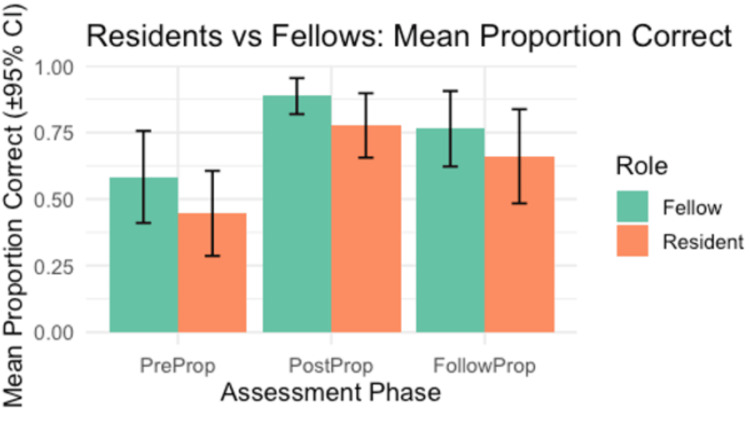
Mean proportion of correct responses (±95% confidence interval) for fellows (green) and residents (orange) across three assessment phases: pre-intervention (PreProp), immediate post-intervention (PostProp), and follow-up (FollowProp). Fellows scored higher than residents at each phase, with both groups demonstrating improvement from pre- to post-assessment and partial retention at follow-up. Differences between groups were statistically significant at the post-intervention phase (t(13)=−3.95, p=.002, d=1.05), indicating a large effect size.

Following implementation of the ultrasound-guided regional anesthesia workshop, participants demonstrated significant improvement in mean proportion correct scores. The mean proportion of correct of the fellows improved from 0.58±0.16 pre-workshop to 0.89±0.09 post-workshop (t(13)=−3.95, p=0.002, d=1.05, large effect) and remained higher at follow-up (t(13)=−2.65, p=.020, d=0.59, medium). Similarly, residents showed significant improvement from 0.45±0.21 pre-workshop to 0.78±0.12 post-workshop (t(13)=−4.01, p=0.001, d=1.21, large) with sustained gains at follow-up (t(13)=−3.12, p=0.008, d=0.66, medium). When all participants were analyzed together, performance improved significantly from pre- to post-workshop (t(27)=−5.72, p<0.001, d=1.15, large) and remained elevated at follow-up (t(27)=−4.15, p<0.001, d=0.63, medium). After Bonferroni correction (adjusted α=.025), all pre- to post-workshop and pre- to follow-up differences remained significant, confirming robust and durable knowledge gains across trainee levels. This data are visually represented by Figure 6.

To assess differences in post-workshop performance between training levels, post-test scores were compared between fellows and residents. Mean percent correct did not differ significantly (t(20.41)=1.56, p=0.13, 95% CI (−0.04, 0.26)); and a Wilcoxon rank-sum test yielded consistent findings (p=0.25). The between-group effect size was large (d=1.05, 95% CI (0.37, 1.74)), indicating comparable post-workshop performance and supporting the curriculum’s applicability across different stages of training. Statistical data are summarized in Table 1.

**Table 1:** Summary of within-group comparisons showing significant improvement in mean proportion correct from pre- to post- and pre- to follow-up assessments with large effect sizes across groups (Bonferroni-adjusted α=0.025).

Group	Comparison	t value	df	p value	p_adj	Cohen’s d	Magnitude
Fellow	Pre vs Post	-3.945	13	0.002	0.010	1.053	Large
Fellow	Pre vs Follow-up	-2.645	13	0.020	0.121	0.589	Medium
Resident	Pre vs Post	-4.006	13	0.001	0.009	1.208	Large
Resident	Pre vs Follow-up	-3.122	13	0.008	0.049	0.662	Medium
Combined	Pre vs Post	-5.721	27	0.000	0.000	1.152	Large
Combined	Pre vs Follow-up	-4.147	27	0.000	0.002	0.632	Medium

At the completion of the in-person workshop, all 20 participants (100%) successfully demonstrated proficiency in obtaining the six critical neuraxial USGA views and relevant sonanatomic landmarks, as verified by instructors utilizing a structured qualitative assessment tool (Figure 3). Each participant completed the task within the allotted three-minute assessment window. Inability to complete the task in three minutes would have been considered a failure.

**Figure 7:**
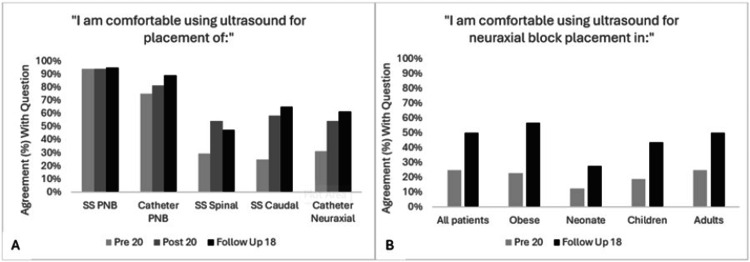
Results of residents, fellows, and combined cohort comfort with using ultrasound for specific blocks (A) and in different patient populations (B) showing improved comfort on the post-quiz and/or six+ month follow-up quiz after participation in the workshop. SS=single shot; PNB=peripheral nerve block.

**Figure 8:**
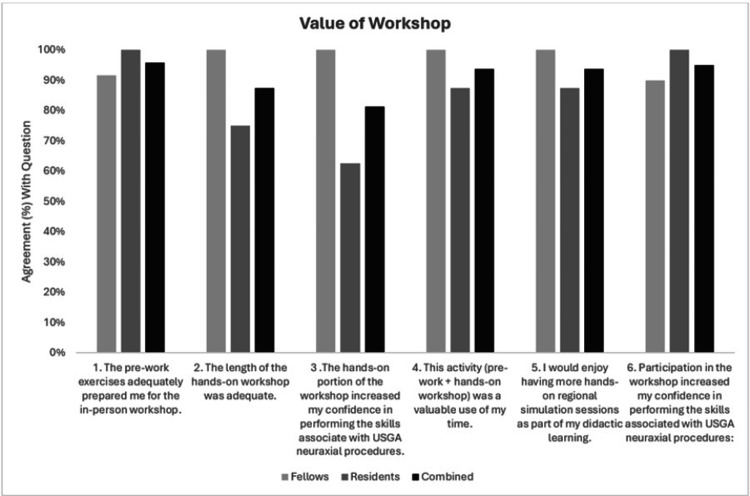
Responses from residents, fellows, and the combined cohort to six questions regarding the components of the workshop and their overall educational value. Questions 1-5 are from the post-quiz and consist of responses from 12 fellows, eight residents, and 20 combined responses. Question 6 is from the six+-month follow up quiz, which had responses from 10 fellows, eight residents, and 18 combined responses.

Self-reported comfort with neuraxial ultrasound increased across all groups, with higher proportions of participants endorsing “somewhat agree” or “strongly agree” and “often” or “always” in the post- and follow-up assessments. Improvements were noted in comfort with ultrasound-guided placement of single-shot spinal, single-shot caudal, and neuraxial catheters, as well as across patient populations including neonates, children, adults, and individuals with obesity (Figures 7A, 7B, respectively). Behavioral intent also shifted positively. Responses to the post-intervention question, “I routinely use ultrasound for neuraxial block placement in all patients, obese, neonate, children, adults,” skewed more favorably at follow-up compared to baseline. Participant feedback was overwhelmingly positive, with strong agreement reported across all six feedback items (Figure 8).

## Discussion

There is increasing evidence supporting the benefit of using ultrasound (US) for neuraxial procedures, especially in pediatric anesthesia [10,11,20-23]. The use of phantoms and simulation for teaching and assessing regional anesthesia techniques is well-established and offers a low-risk setting for trainees to engage in opportunities to learn procedures that would otherwise involve high-risk in actual patients [2,3,6-8]. However, current literature lacks information on phantom models and teaching curricula specific to these populations.

Our findings demonstrate that a structured hands-on curriculum, when combined with a validated ballistics gel spine phantom [18], enables pediatric anesthesia trainees to both acquire and retain the knowledge, at six or more months, required to obtain the six critical ultrasound views necessary for successful placement of neuraxial anesthesia. Furthermore, we established that the phantom could be used both for skills education and for skills assessment. Not only did the training enhance knowledge and technical skills, but it also improved trainees’ confidence and translated into measurable behavioral change, with ultrasound guidance for neuraxial procedures shifting from rare to more routine practice. Although comfort is inherently subjective, these gains paralleled objectively verified scanning competence demonstrated during the workshop. Trainees rated the workshop highly and expressed strong support for the broader integration of similar educational interventions into pediatric anesthesiology training.

A major strength of this approach is its affordability and reproducibility. The ballistics gel phantom can be constructed at low cost using readily available materials, making it accessible to programs regardless of resource limitations. Each phantom costs approximately $150 to construct using locally available materials, as previously described [18], making replication feasible even in low-resource training environments. Unlike high-fidelity commercial phantoms, which are prohibitively expensive and often not tailored for pediatric applications, our model is cost-effective, durable, and easily replicated, allowing institutions to incorporate it into curricula without substantial financial barriers. Furthermore, the curriculum itself is highly adaptable, making it generalizable across a wide range of training environments, from large academic centers to smaller residency programs with fewer pediatric cases.

Though our results are overall positive, there are several limitations to our study. First, we did not use a validated regional anesthesia assessment tool, such as those described by Chuan et al. [24], and instead created our own for the hands-on portion of the workshop. The major reason for not doing so is that most focus on the hand-eye coordination skills related to needle passes in the performance of regional anesthetic procedures rather than on the ability to obtain necessary ultrasound imaging for executing regional blocks. As we did not have trainees perform needling techniques in this study, such assessment tools did not apply. While a formal tool was not employed, our qualitative checklist was designed to align with key domains emphasized in the American Society of Regional Anesthesia (ASRA) and other competency-based evaluation frameworks. Ideally, trainees would have had another skills assessment repeated at the six-month follow up. Unfortunately, due to the one-year fellowship and constraints on timing of the workshop, many fellows had graduated and left for other institutions at follow-up. Presenting the workshop earlier in the fellowship year would abate this problem.

Another limitation was the inclusion of residents very early in their Clinical Anesthesia Year 2 (CA-2) without adaptation of the curriculum for their stage of training, compared to the more experienced fellows. Some residents in the CA-2 group had not yet placed any epidural or spinal blocks, even by anatomic landmark technique, and therefore had much less background or context for the use of USGA in neuraxial techniques. The degree of inexperience in the CA-2 group likely contributed to the lower percent correct and improvement on the post-quiz knowledge-based questions. However, adapting the curriculum would not have allowed for direct comparison of results. Nonetheless, residents did demonstrate significant gains in knowledge and the equal ability to demonstrate scanning skills taught in the workshop.

Additionally, while comfort and behavioral questions show positive trends over time, they may reflect both the effects of the workshop and continued clinical exposure to USGA techniques over the training year. To make some distinction with ultrasound experience, questions regarding comfort with single shot (SS) and catheter-based peripheral nerve blocks (PNB) were included. As seen in Figure 7, comfort levels started and remained high for SS PNB, as well as catheter PNB. When compared to the neuraxial procedures, comfort started much lower and appeared to increase more significantly after the workshop, at both time points. While immediate improvements in comfort could be attributed to the workshop, increased comfort at follow up could have been due to gains in clinical experience. Including a control group, who received only the quizzes and no intervention, would have strengthened our findings and perhaps allowed us to decipher more definitively whether the workshop itself, versus increased time in training, directly contributed to the improved comfort and behavioral changes noted.

Furthermore, while we demonstrated improvement in knowledge, technical scanning ability, and self-reported comfort, we did not directly evaluate the effect of this training on clinical learning curves, procedural success rates, or patient outcomes. These results should therefore be interpreted as evidence of effective foundational training in a simulated environment. Additional research, focusing on longitudinal tracking of procedural success or complication rates post-training, is needed to assess transferability to real-world practice. However, incorporating real patient scanning would have required added consent and workflow modifications that were beyond the scope of this feasibility study. Future work should examine whether phantom-based training shortens the clinical learning curve and should directly compare phantom and patient scanning to establish real-world impact.

Finally, participant attrition occurred across study phases. Because this was a voluntary educational project without incentives or protected time, several residents did not complete post-workshop assessments, and two fellows were lost to follow-up after graduation. As participation was voluntary, a degree of selection bias is possible and may influence the generalizability of these results. Although attrition reduced the number of paired data points, the remaining cohort still exceeded our a priori power calculation, and statistically significant improvements were observed across groups.

There are multiple next steps and applications that can be pursued for research and quality improvement related to creation of our phantom-based workshop curriculum. Our curriculum provides a framework for other programs to enhance their simulation-based teaching. Also, evaluating the effectiveness of the phantom and curriculum for experienced clinicians - who may be highly proficient in landmark-based techniques but less familiar with USGA methods - represents an important opportunity. Designing phantoms with unusual spinal anatomy, including scoliosis or tethered cord for example, could further serve clinician’s educational needs in the ability to recognize and identify abnormalities that may require optimization or abortion of planned neuraxial procedures, perhaps saving patients from neuraxial attempts that are likely to fail. From a quality and safety standpoint, data suggests that first pass and overall success rate of neuraxial procedures, specifically single shot spinal and/or caudal epidural blocks in infants and children, is improved with the use of ultrasound [10,11]. Collecting patient-level data on success rates before and after workshop completion could help ascertain whether there are measurable clinical benefits to patients.

## Conclusions

There exists a growing need for simulation education in USGA skills, especially as the usefulness of ultrasound has become more apparent broadly and in its application to the performance of regional and neuraxial anesthesia in pediatric patients. This study demonstrates that a structured, simulation-based curriculum in pediatric regional anesthesia can significantly improve and sustain trainee knowledge across learner levels. Both residents and fellows achieved meaningful gains in procedural understanding, with large pre- to post-workshop effect sizes and durable retention at follow-up. The consistent results across groups underscore the adaptability of this low-cost, reproducible educational model. By emphasizing deliberate practice and faculty-standardized assessment, this curriculum addresses recognized training gaps in pediatric regional anesthesia education. Future multicenter validation will be essential to assess generalizability across diverse training environments and to evaluate long-term translation of these skills into clinical performance.
